# Chemokine expression in peripheral tissues from the Monosodium Iodoacetate model of chronic joint pain

**DOI:** 10.1186/1744-8069-9-57

**Published:** 2013-11-08

**Authors:** John M Dawes, Hannes Kiesewetter, James R Perkins, David LH Bennett, Stephen B McMahon

**Affiliations:** 1 Nuffield Department of Clinical Neurosciences, West Wing, Level 6, John Radcliffe Hospital, Oxford OX3 9DU, UK; 2 Wolfson Centre for Age-Related Disease, King’s College London, London, UK; 3 Department of Structural and Molecular Biology, University College London, London, UK

**Keywords:** Pain, Chemokine, Osteoarthritis, Monosodium iodoacetate, Macrophages, Neutrophils

## Abstract

**Background:**

Chronic pain arising from degenerative diseases of the joint such as osteoarthritis (OA) has a strong peripheral component which is likely to be mediator driven. Current treatments which reduce the production of such mediators i.e. non-steroidal anti-inflammatory drugs (NSAIDs), can help to lessen pain in OA patients. However, this is not always the case and complete pain relief is rarely achieved, suggesting that additional unidentified mediators play a role. Here we have investigated the notion that chemokines might act as such pain mediators in OA.

**Results:**

Using the monosodium iodoacetate (MIA) model of chronic joint pain the expression of over 90 different inflammatory mediators, mainly cytokines and chemokines, were measured in tissues taken from the femorotibial joint (cartilage, subchondral bone, fat pad) using custom-made quantitative real-time polymerase chain reaction (qPCR) array cards. At both the day 3 and 14 time points, numerous inflammatory mediators were significantly up-regulated in these tissues, although it was clear that the largest transcriptional dysregulation occurred in the cartilage. Using individual qPCR to measure immune cell markers, a significant infiltration of macrophages was measured in the cartilage and fat pad at day 3. Neutrophil infiltration was also measured in the fat pad at the same time point, but no infiltration was observed at day 14. Combination of mRNA expression data from different time points and tissues identified the chemokines, CCL2, 7 and 9 as being consistently up-regulated. The overall increase in CCL2 expression was also measured at the protein level.

**Conclusion:**

Chemokines in general and CCL2, 7 and 9 in particular, represent promising targets for further studies into the identification of new pain mediators in chronic joint pain.

## Background

The exact aetiology of osteoarthritis (OA) is still unclear, but is likely driven by an over-active chondrocyte population within the cartilage of the affected joint [1]. Eventually atypical chondrocytes favour a catabolic phenotype, releasing many factors, such as cytokines and chemokines, which can promote the degradation process by increasing the production of matrix degrading enzymes [2-4]. Further progression leads to the degradation of the articular cartilage as well as the underlying subchondral bone, with bouts of synovitis.

Pain is the most common symptom of OA and often used as a criterion for diagnosis. Interestingly, it seems that there is a strong peripheral drive to the persistent pain experienced by OA patients. For example, the intra-articular injection of local anaesthetics can significantly reduce pain scores [5] and the surgical removal of the diseased joint often leads to the complete ablation of pathological pain [5,6]. NSAIDs represent the first line analgesic treatment and are effective when given topically [7]. Therefore it is likely that pro-algesic mediators in the periphery make a strong contribution. However, NSAIDs are often unable to completely relieve OA pain [8], unlike joint replacement where all putative peripheral pain mediators are removed, and such observations suggest additional pain mediators have a role. For instance, recent work has shown that the neutralisation of the pro-algesic mediator nerve growth factor is analgesic in OA patients [9]. In addition prolonged NSAID use commonly causes severe adverse effects [10] and therefore the identification of unrecognised pain mediators might allow for the development of more adequate pain therapies with reduced side-effect profiles.

Cytokines and chemokines have themselves been implicated in modulating pain processing [11]. The peripheral application of members such as IL1β, TNFα, CCL2 and CXCL1 can induce pain-related behaviours in rodents [12-14]. Since these same factors are present in the osteoarthritic joint, they represent an interesting group of potential pain mediators in OA.

The cartilage is a key tissue in the generation of inflammatory factors during OA [4]. Despite evidence showing that the cartilage can become innervated during joint degradation [15], this tissue is generally considered to be aneural and therefore it is unclear as to how much pro-algesic molecules produced here might contribute to chronic joint pain. Other peri-articular tissues such as the subchondral bone and synovium are highly innervated [16,17], as well as the rarely considered infrapatellar fat pad [18], and might represent more important pain producing sites in OA.

Using the chemically-induced, monosodium iodoacetate (MIA) model of chronic joint pain, in which histological signs reflect some of those seen clinically [19-22], the expression of both chemokines and cytokines have been measured using custom-made qPCR array cards. This was carried out in cartilage, subchondral bone and fat pad, with the aim of identifying mediators which may participate in driving OA pain.

## Results

### Time course of pain-related behaviour in the MIA model

**Figure 1:**
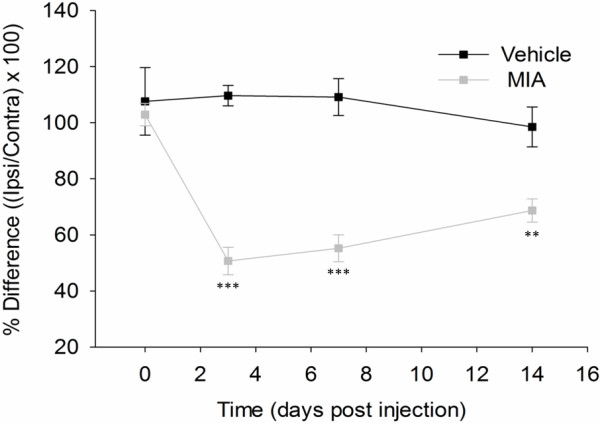
**Time course of primary pain-related hypersensitivity in the MIA model.** Significant primary pain-related hypersensitivity in the MIA model, 3, 7 and 14 days post induction compared to vehicle treated animals as measured by the percentage of weight bearing asymmetry between ipsilateral and contralateral hind limbs. Two-way repeated measures ANOVA, post hoc Tukey, **p < 0.01, ***p < 0.001; n = 6. All data are expressed as mean ± SEM.

Pain-related behaviour is a well-established feature of the MIA model and has previously been demonstrated in a number of species [22-25]. Weight bearing asymmetry is considered a measure of primary pain-related behaviour in the MIA model and is reduced by treatment with analgesics such as NSAIDs [19,22,26,27]. Here, the presence of such pain-like behaviour was confirmed after intra-articular injection of 1 mg MIA (Figure 1). Figure 1 shows a significant reduction in weight borne on the ipsilateral limb in the MIA model at days 3, 7 and 14 when compared to vehicle treated rats. The greatest deficient was measured at day 3 with ipsilateral hind limb weight bearing in the MIA model on average reduced to 50.7 ± 4.9%, compared to 109.7 ± 3.6% in vehicle treated animals. The mean weight borne on the ipsilateral hind limb increased at day 14 (68.7 ± 4.1%) but still remained highly significantly different when compared to control animals (98.5 ± 7.1%). This data confirms the presence of primary pain-related behaviour in the MIA model at both day 3 and 14.

### Regulation of inflammatory mediators in femorotibial joint tissues from the MIA model

Using custom-made qPCR array cards the relative expression levels of 92 different target transcripts were measured in the MIA model from cartilage, subchondral bone and the infra-patellar fat pad, and compared to vehicle treated rats at both day 3 and 14 post induction. It should be noted that the subchondral bone samples used for RNA extraction also contained the underlying bone marrow. All qPCR array data from each time point and tissue are shown in Additional file 1: Table S1. Data are displayed as the mean fold change (FC) which is the relative abundance of the transcript in the MIA model in comparison to vehicle treated animals when setting the level in these animals as 1.0. The range of one standard deviation from this mean is shown in parenthesis. Of particular interest were those transcripts most up-regulated that could potentially be driving the abnormal pain-like behaviours. A biological significance was arbitrarily defined as a greater than 2 FC in the relative expression of the transcript in MIA treated tissue when compared to the expression in control.

### Gene expression changes: Day 3

Three days after injury a number of transcripts were dysregulated in the cartilage, fat pad and subchondral bone of the MIA model when compared to vehicle control animals. Table 1 shows the top twelve up-regulated transcripts ranked in order of FC for the each tissue. In the cartilage CCL21 (20.8 (4.0-108.2)) showed the greatest fold increase compared to control, although this change was not statistically significant. However, four genes in the top twelve were significantly increased in the MIA model for cartilage; the chemokines CCL12 (13.9 (6.0-31.9)), XCL1 (4.4 (3.8 – 5.1)) and CCL5 (3.9 (2.9 – 5.4)), and the macrophage marker ionised calcium-binding adapter molecule 1 (IBA1) (3.6 (2.8 – 4.6)). As with the cartilage, all genes in the top 12 for the subchondral bone data set showed a greater than 2-fold increase in their expression. Here the top up-regulated gene, CCL17 (5.7 (4.5-7.3)), was of a smaller magnitude of increase when compared to the top gene in cartilage, but was statistically significant versus expression in the vehicle group. Another chemokine CCL9 (2.4 (1.9-3.0)) was also statistically significantly increased in the subchondral bone at day 3. In the infrapatellar fat pad, not all of the top 12 transcripts shown had a greater than 2-fold increase in their expression following MIA treatment, unlike in the cartilage and bone. Still, there were a number of significant changes in the fat pad and these included the chemokine CXCL5 (4.9 (3.2-7.6)), inducible nitric oxide synthase (iNOS, 5.0 (2.9-8.8)) and a member of the epidermal growth factor receptor (EGFR) ligand family, amphiregulin (AREG, 7.0 (3.8-13.2)).

**Table 1:** DAY 3: Top up-regulated inflammatory mediators in the MIA model

**Rank**	**Cartilage**		**Subchondral bone**		**Fat pad**	
	**Gene name**	**FC**	**Gene name**	**FC**	**Gene name**	**FC**
1	CCL21	20.8 (4.0-108.2)	CCL17	5.7* (4.5-7.3)	EREG	10.1 (2.8-36.3)
2	IL12B	13.9 (3.7-51.9)	IL12b	5.4 (2.3-12.8)	NRG1	7.4 (2.6-20.9)
3	CCL12	13.9* (6.0-31.9)	BDNF	4.8 (1.8-13.1)	AREG	7.0* (3.8-13.2)
4	EREG	8.3 (2.6-25.7)	CCL7	4.7 (2.6-8.4)	iNOS	5.0* (2.9-8.8)
5	CCL2	5.8 (2.7-12.6)	CXCL11	4.3 (1.4-13.1)	CXCL5	4.9* (3.2-7.6)
6	CCL7	5.8 (2.8-11.7)	IL6	3.5 (1.0-12.0)	GCSF	4.1 (1.7-10.1)
7	CXCL1	4.9 (2.3-10.2)	CCL2	2.9 (1.7-5.0)	IL11	3.8 (2.2-6.4)
8	XCL1	4.4** (3.8-5.1)	CCL5	2.6 (1.6-4.2)	CCL3	2.3 (-1.3-7.2)
9	CCL5	3.9* (2.9-5.4)	EDN1	2.5 (1.2-4.9)	ARTN	2.3 (1.6-3.3)
10	CXCL13	3.7 (1.3-10.3)	ARTN	2.4 (1.4-4.4)	LIF	2.1 (1.4-3.1)
11	IBA1	3.6* (2.8-4.6)	CCL9	2.4* (1.9-3.0)	NGF	1.9 (1.4-2.5)
12	CXCL2	3.5 (-1.3-15.1)	CCL21	2.3 (-4.9-26.2)	CXCL17	1.9 (-1.5-5.4)

*p<0.05, *p<0.01. CCL; chemokine (CC-motif) ligand. IL; Interleukin. EREG; Epiregulin. CXCL; chemokine (CXC-motif) ligand. XCL; chemokine (XC-motif) ligand. IBA1; ionised calcium binding adaptor molecule 1. BDNF; Brain derived neurotrophic factor. EDN1; Endothelin 1 ARTN; Artemin. NRG1; Neuregulin 1. AREG; Amphiregulin. iNOS; inducible nitric oxide. GCSF; Granulocyte colony stimulating factor. LIF; Leukemia inhibitory factor. NGF; Nerve growth factor.

**Figure 2:**
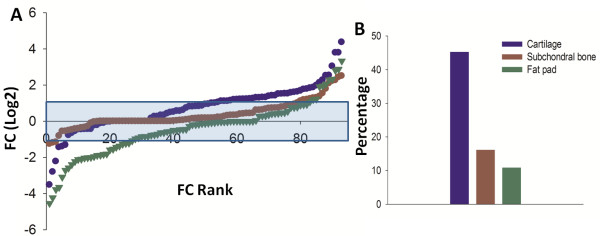
**Day 3: Distribution of inflammatory mediator expression in each tissue as ranked by FC. (A)** The distribution profile of mediator expression in each tissue at day 3 in the MIA model. The data points show the mean FC for each individual transcript. These values are ranked in order, starting from those most down-regulated (compared to control), to those most up-regulated. Adjacent data points in different tissues do not necessarily correspond to the same gene. The blue shaded box represents an area of +/-2 ≤ FC. X-axis is on a log2 scale. Data are displayed as the mean FC, FC = MIA/Control, n = 92. **(B)** The percentage of genes > 2 FC up-regulated in the each tissue. As shown, the greatest percentage of genes was up-regulated in the cartilage (42.5%) as compared to the subchondral bone (16.1%) and fat pad (10.8%).

To gain a better overall idea of inflammatory mediator expression change in the MIA model at day 3, the distribution of gene expression for each tissue is shown in Figure 2A ranked in order of FC. Overall for each tissue the majority of genes were not dysregulated by MIA injection at day 3 (blue shaded area). However, as highlighted in Table 1, there were a group of genes in each tissue, which were up-regulated when compared to control, although the number in each tissue was different. Since the femorotibial joint is comprised of many tissues which are affected during OA disease, it is likely that they could all contribute to the production of putative pain mediators. Therefore, the identification of the principal mediator producing tissue might be important from a pain perspective. In the subchondral bone and fat pad up-regulated transcripts amounted to 16.1% and 10.8%, respectively, of the total genes measured (Figure 2B). This is in contrast to the cartilage where 42.5% of inflammatory mediator transcripts were up-regulated. In addition, these genes were generally of a greater magnitude of FC when compared to the other tissues studied. Therefore in terms of inflammatory mediator expression, the cartilage seems to be the main contributor at day 3 in the MIA model. It should be noted that a number of genes were also down-regulated, with the majority seen in the fat pad.

### Gene expression changes: Day 14

In the MIA model 14 days after induction, the same transcripts were measured from the same femorotibial joint tissues. Table 2 shows the top twelve up-regulated transcripts ranked in order of FC for the each tissue. At day 14 it seems that of the top 12 transcripts, the majority were significantly increased when compared to control levels. Of particular interest were the three top up-regulated genes, CCL7 (25.5 (12.1 – 53.8)), CCL2 (21.8 (12.9 – 37.0)) and CCL9 (17.0 (12.5 – 23.0)). All of these were up-regulated at day 3, but their magnitude of fold increase was much greater 14 days after MIA injection. In addition, the chemokine CCL17 (10.5 (5.1 – 21.5)), the top ranked transcript in the subchondral bone at day 3, was now significantly up-regulated in the cartilage. The subchondral bone had fewer genes up-regulated (only 8 of the top 12), although the top genes were of a greater magnitude of change, highlighted by CCL9 which was now significantly increased by 10.8 (7.1-16.5) fold compared to control. Although genes in the fat pad also showed a greater magnitude of change, none of these were significant.

**Table 2:** DAY 14: Top up-regulated inflammatory mediators in the MIA model

**Rank**	**Cartilage**		**Subchondral bone**		**Fat pad**	
	**Gene name**	**FC**	**Gene name**	**FC**	**Gene name**	**FC**
1	CCL7	25.5* (12.1-53.8)	CCL9	10.8* (7.1-16.5)	BDNF	26.1 (11.1-61.4)
2	CCL2	21.8** (12.9-37.0)	CCL7	7.2 (1.6-31.6)	iNOS	10.6 (2.7-40.8)
3	CCL9	17.0** (12.5-23.0)	CCL22	6.3 (3.2-12.6)	CCL1	10.1 (2.3-44.0)
4	EDN1	13.7 (5.1-37.2)	CCL2	3.7 (2.2-6.1)	IL3	4.0 (1.0-15.7)
5	iNOS	11.4* (8.1-16.2)	LIF	3.6 (2.7-4.7)	IL27B	4.0 (2.0-7.8)
6	CCL17	10.5* (5.1-21.5)	CCL17	2.9 (-1.1-9.1)	CCL21	3.9 (1.9-8.2)
7	AREG	8.1 (2.7-24.3)	IL11	2.6 (1.1-6.5)	AREG	3.8 (2.6-5.8)
8	IL11	6.8 (3.7-12.5)	KITLG	2.2 (1.2-4.1)	CXCL5	2.9 (1.6-5.4)
9	CXCL13	6.6 (2.4-18.3)	iNOS	1.9 (1.2-3.1)	IL1A	2.8 (-1.7-12.6)
10	PTGES	5.8** (4.8-7.0)	IL1A	1.9 (-1.0-3.7)	CCL9	2.7 (1.7-42)
11	CCL22	5.7* (3.4-9.4)	ARTN	1.8 (1.1-3.0)	NRG1	1.9 (-3.3-12.3)
12	CCL12	5.0 (2.7-9.2)	IL1B	1.7 (1.1-2.7)	IL7	1.8 (-1.4-4.6)

*p<0.05, **p<0.01 CCL; chemokine (CC-motif) ligand. IL; Interleukin EDN1; Endothelin 1. iNOS; inducible nitric oxide. AREG; Amphiregulin. CXCL; chemokine (CXC-motif) ligand. PTGES; Prostaglandin E synthase. LIF; Leukemia inhibitory. KITLG; c-KIT ligand. ARTN; Artemin. BDNF; Brain derived neurotrophic factor. NRG1; Neuregulin 1*.*

**Figure 3:**
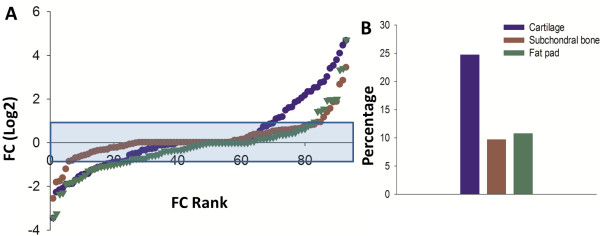
**Day 14: Distribution of inflammatory mediator expression in each tissue as ranked by FC. (A)** The distribution profile of mediator expression in each tissue at day 14 in the MIA model. The data points show the mean FC for each individual transcript. These values are ranked in order, starting from those most down-regulated (compared to control), to those most up-regulated. Adjacent data points in different tissues do not necessarily correspond to the same gene. The blue shaded box represents an area of +/-2 ≤ FC. X-axis is on a log2 scale. Data are displayed as the mean FC, FC = MIA/Control, n = 92. **(B)** The percentage of genes > 2 FC up-regulated in the each tissue. As shown, the greatest percentage of genes was up-regulated in the cartilage (24.7%) as compared to the subchondral bone (9.7%) and fat pad (10.8%).

The distribution of gene expression for each tissue at day 14 is shown in Figure 3A. Here there is a striking increase in the expression of inflammatory mediators in the cartilage when compared to the other tissues. With the exception of the top up-regulated factor in the fat pad, the increase in transcription is also of a much greater magnitude in the cartilage. The profile of up-regulated genes in the fat pad and subchondral bone seem very similar and in comparison to day 3 the percentage of genes up-regulated was the same in the fat pad, 10.8%, and slightly decreased in the bone to 9.7% (Figure 3B). In contrast, the percentage of up-regulated genes in the cartilage was clearly decreased to 24.7% (Figure 3B). In line with expression changes measured at day 3 in the MIA model, the greatest transcriptional up-regulation seems to occur again in the cartilage. Although the percentage of genes overexpressed was lower at day 14 than at day 3, the magnitude of fold increase was much greater.

### Identifying putative pain mediators

**Figure 4:**
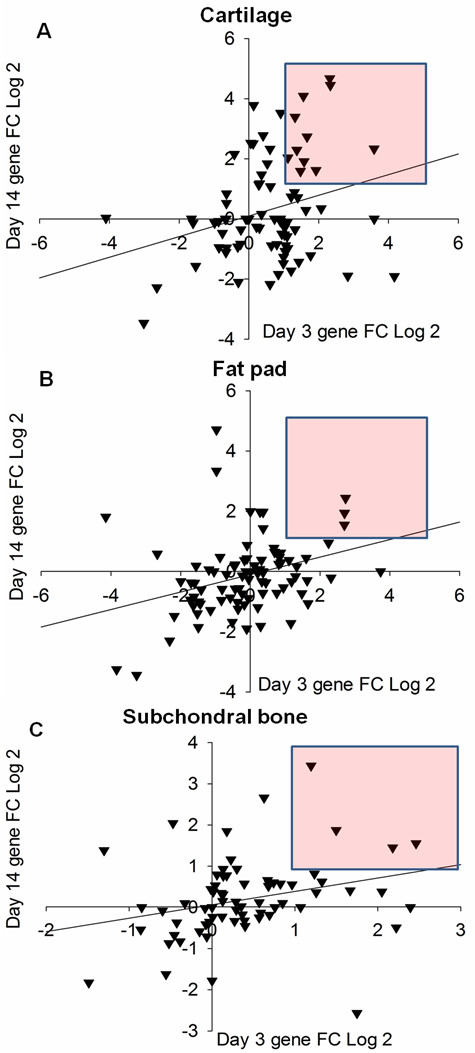
**Correlation of inflammatory mediator expression between day 3 and 14 in each femorotibial tissue.** Day 3 and day 14 gene expression was plotted against each other for the cartilage **(A)**, fat pad **(B)** and subchondral bone **(C)**. For each tissue there was a positive and significant correlation (A-C, r = 0.227 p = 0.00743, r = 0.327 p = 0.00147, r = 0.283 p = 0.00629, respectively). By expressing the data this way, those factors which had a > 2 fold increase at both time points could be identified (red shaded area). In total there were 11 of these genes in the cartilage **(A)**, 3 in the fat pad **(B)** and 4 in the subchondral bone **(C)**. Pearson’s correlation coefficient, n = 92. Data is displayed as the mean FC, FC = MIA/Control. Both axes on Log 2 scale.

From the data presented it is clear that the cartilage experienced the greatest transcriptional dysregulation at both time points. It is, however, unclear as to what effect time had on individual mediator expression. In terms of recognizing putative pain mediators, it seems important to identify those genes up-regulated at both day 3 and 14 in each tissue, since both of these time points were associated with robust pain-related behaviour (Figure 1). Figure 4 shows that the correlation of gene expression for each tissue between day 3 and 14 occurred in a positive and significant manner (Figure 4A-C, r = 0.227 p = 0.00743, r = 0.327 p = 0.00147, r = 0.283 p = 0.00629, respectively). These correlations do imply that the same mediators might be driving pain-like behaviours at both time points. The red shaded area shows the genes with a greater than 2-fold increase at both day 3 and 14. This related to 12 genes in the cartilage (Figure 4A), 3 in the fat pad (Figure 4B) and 4 in the subchondral bone (Figure 4C). These genes are listed in Table 3 and it is evident that the majority are members of the chemokine family. It should be noted that the genes consistently up-regulated in the subchondral bone, were also up-regulated in the cartilage at 3 and 14 days post MIA induction. This observation indicates that the same mediators were also increased in different tissues. To combine tissue data at each time point a combined ranking approach was used (see Methods) where each gene received a combined rank value (CRV). Within each data set genes were ranked by FC and received a rank value depending on their position. These rank values were then averaged to obtain a CRV. This approach allowed for an accurate method for quantifying rank across data sets and gave an idea of rank variability. The top ranked genes, i.e. those genes that were consistently up-regulated across tissues, are shown for both day 3 and 14 also in Table 3. At day 3, 8 of the top 12 combined ranked genes were chemokines and 7 of these were members of the same subfamily, with CCL7 the overall top ranked gene. At day 14 the chemokine CCL9 was the top overall ranked gene. Here non-chemokine transcripts make up half of the top combined ranked factors, with iNOS being the second top ranked gene at day 14. More bluntly, combined ranking was used to combine all data sets and this is shown in the last column in Table 3. When all of these comparisons are considered it is easy to see that some genes were consistently up-regulated across tissues and time points. For example, iNOS was found in four of the comparisons made and there is evidence to suggest that this enzyme, which is primarily found in immune cells, has a role in pain processing in general [28] and might even regulate OA pain [29]. In contrast, the up-regulation of the pro-algesic factor CXCL5 [30] was restricted to the fat pad, suggesting that putative OA pain mediators might be tissue constrained.

**Table 3:** Top ranked mediators across different comparisons in the MIA model

**Rank**	**Greater than 2 FC**			**Combined ranking**					
	**Cartilage**	**Subchondral bone**	**Fat pad**	**Day 3**	**CRV (±SEM)**	**Day 14**	**CRV (±SEM)**	**Day 3 + 14**	**CRV (±SEM)**
1	CCL2	CCL7	iNOS	CCL7	83.0 (5.0)	CCL9	88.3 (2.7)	CCL7	85.0 (3.2)
2	CCL7	CCL17	AREG	CCL5	81.3 (3.2)	iNOS	87.7 (2.0)	CCL2	83.3 (3.8)
3	CCL12	CCL2	CXCL5	CCL2	80.0 (7.0)	CCL7	87.0 (4.5)	CCL9	80.5 (5.3)
4	CCL9	CCL9	-	CCL3	79.0 (2.9)	CCL2	86.7 (3.4)	ARTN	78.2 (2.4)
5	CXCL13	-	-	XCL1	78.3 (4.4)	BDNF	80.7 (5.8)	iNOS	77.8 (6.5)
6	CCL17	-	-	ARTN	78.0 (5.0)	ARTN	78.3 (2.0)	IL18	69.3 (3.9)
7	AREG	-	-	IL6	77.0 (5.8)	IL1A	78.3 (5.2)	AREG	66.2 (12.8)
8	XCL1	-	-	EREG	76.3 (14.2)	CCL22	78.0 (8.3)	XCL1	65.5 (10.6)
9	CXCL2	-	-	CCL21	76.0 (11.0)	CXCL16	72.3 (1.3)	IBA1	65.2 (5.5)
10	IL18	-	-	CCL12	73.3 (11.6)	IL18	72.0 (1.5)	CCL21	64.7 (13.0)
11	ARTN	-	-	PROK2	73.3 (4.2)	CCL4	71.7 (3.2)	CCL4	64.5 (6.9)
12	iNOS	-	-	CCL9	72.7 (8.4)	IL7	71.7 (5.0)	BDNF	63.7 (13.2)

CRV = combined rank value. CCL; chemokine (CC-motif) ligand. AREG; Amphiregulin. XCL; chemokine (XC-motif) ligand IL; Interleukin. CXCL; chemokine (CXC-motif) ligand. ARTN; Artemin. iNOS; inducible nitric oxide. EREG; Epiregulin. PROK2; Prokineticin 2. BDNF; Brain derived neurotrophic factor. IBA1; ionised calcium binding adaptor molecule 1.

The identification of genes which should be targeted for further investigation is important. An over-representation in Table 3, i.e. an indication of consistent up-regulation, is one way of recognising such factors. It seems obvious that the chemokines CCL2, 7 and 9 fall into this category since all three were listed amongst the top genes in 5 out of the 6 comparisons and were the top 3 ranked genes when all data was combined. CCL2 has previously been implicated in pain; however CCL7 and CCL9 represent novel targets. Therefore these three chemokines seem to signify promising targets for the identification of putative pain mediators in OA and using conventional qPCR the increased mRNA expression of CCL2 and CCL9 has been validated (Additional file 2).

**Figure 5:**
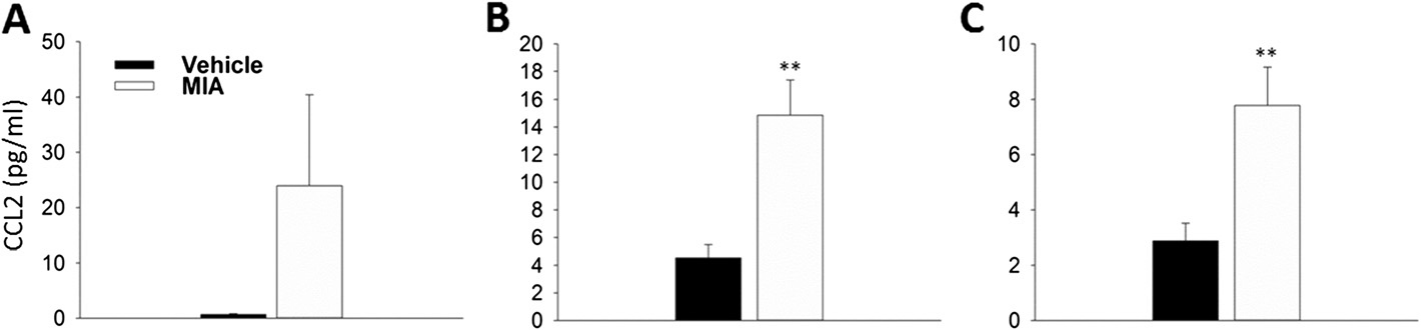
**CCL2 protein expression at day 14 in the MIA model.** The amount of CCL2 protein was measured in vehicle and MIA animals 14 days after treatment in the cartilage **(A)**, subchondral bone **(B)** and fat pad **(C)**. A large increase in CCL2 protein was measured in the cartilage **(A)** although when compared to vehicle treated animals this was not statistically significant. Significant increases in CCL2 protein was measured in the subchondral bone **(B)** and fat pad **(C)**. The protein concentration shown is normalised to 1 mg/ml of total protein. *T*-test, **p < 0.01, n = 3 **(A)**, n = 6 **(B and C)**. All data are expressed as mean ± SEM.

However for these chemokines to truly be targets for future studies into chronic joint pain it is important to validate that the increase in mRNA is also seen at the protein level as this may not always be the case [31]. Here we have used CCL2 as an exemplar. Using protein lysates obtained from cartilage, subchondral bone and fat pad at day 14 in the MIA model, the level of CCL2 was compared against vehicle treated control tissue. Figure 5A shows that, although not statistically significant, there is a large increase in CCL2 protein 14 days after MIA injection in the cartilage. Using neat protein lysates the level of CCL2 protein in vehicle treated cartilage was just within the range of detection and when normalised to 1 mg/ml of the total protein loaded, the average amount was 0.7 ± 0.1 pg/ml. This is in contrast to MIA treated tissue where the average normalised CCL2 protein level detected was 23.9 ± 16.5 pg/ml. For both the subchondral bone and fat pad a significant increase in the level of CCL2 was measured from MIA treated animals versus control (Figure 5B and C). The level of CCL2 in vehicle treated animals was 4.5 ± 0.9 and 2.9 ± 0.6 pg/ml for the subchondral bone and fat pad respectively. This increased to 14.9 ± 2.5 pg/ml in the subchondral bone and 7.8 ± 1.4 pg/ml in the fat pad 14 days after MIA treatment.

### Immune cell infiltration in femorotibial joint tissues of the MIA model

**Figure 6:**
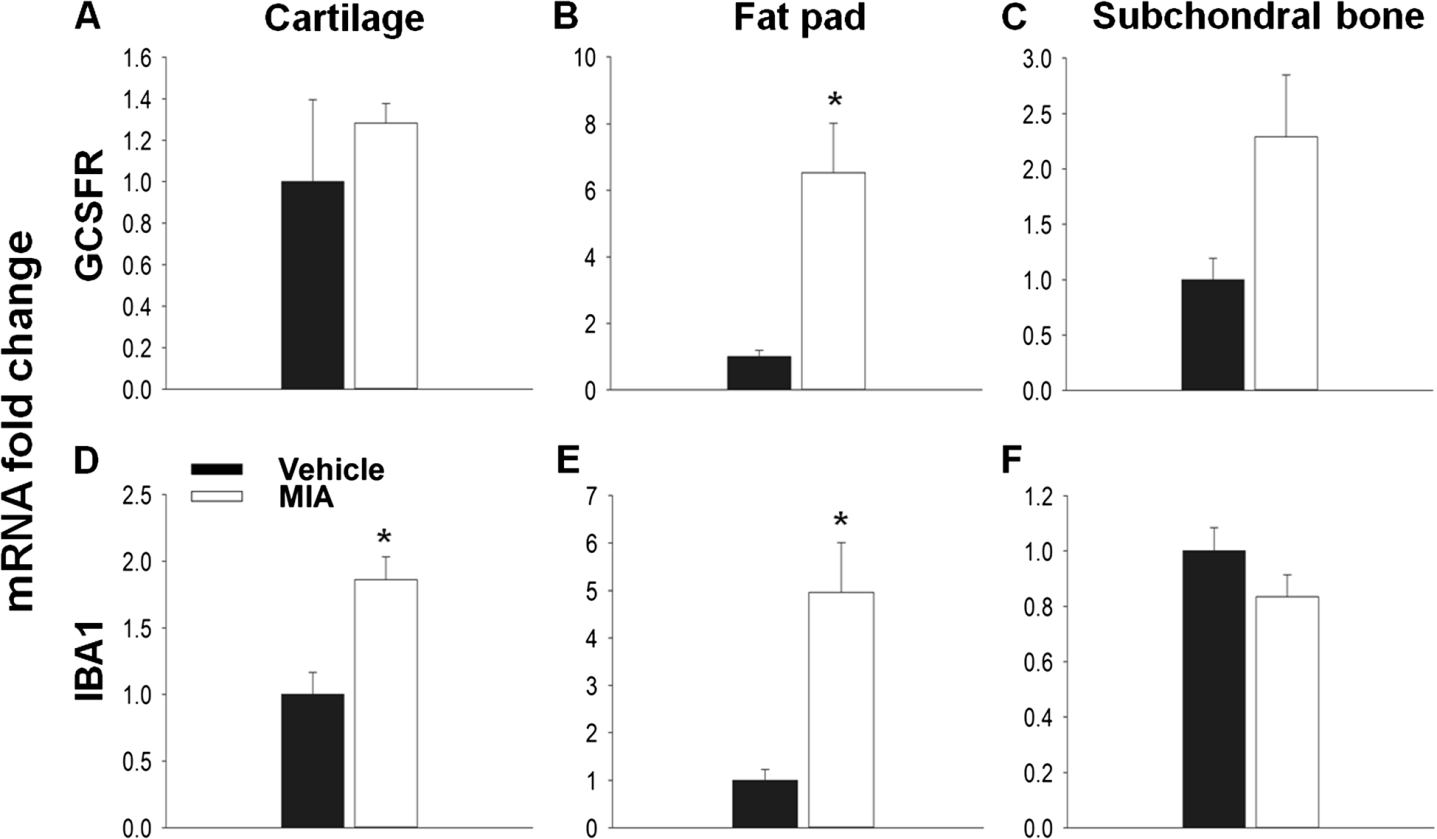
**Day 3: Expression of immune cell markers in the MIA model.** Relative changes in the transcript levels of GCSFR **(A-C)** and IBA **(D-F)** were measured in the MIA model compared to vehicle controls. Here GCSFR and IBA were used to measure neutrophil and macrophage infiltration respectively. No significant change was measured between MIA and controls in both cartilage **(A)** and the subchondral bone **(C)**. However a significant increase in GCSFR expression was measured in the fat pad of MIA treated animals **(B)**. With IBA1 a significant increase in expression was measured in the MIA group in both the cartilage **(D)** and fat pad **(E)**. No difference was measured in the subchondral bone **(F)**. T –Test **(C, D-F)**, Mann–Whitney Rank Sum Test **(A-B)**, *p < 0.05; n = 4. All data are expressed as mean ± SEM.

Chemokines play a pivotal role in the recruitment of immune cells and the orchestration of the inflammatory response, and many of these cell types have been implicated in enhancing pain processing in the periphery through the release of algogenic factors [32]. Therefore cell recruitment is one possible mechanism by which chemokines might contribute to OA pain. The first few days of the MIA model are represented by an initial inflammatory phase [19,21]. Here, using qPCR to measure the expression of the cell markers IBA1 and GCSFR for macrophages and neutrophils respectively, immune cell infiltration was seen in the MIA model at day 3 (Figure 6). Although no significant increase in GCSFR expression was seen in the cartilage (Figure 6A), a significant increase in relative mRNA expression of IBA1 (1.8 ± 0.17) compared to vehicle (1.0 ± 0.17, Figure 5D) was found. In the fat pad both GCSFR (6.5 ± 1.5) and IBA1 (5.0 ± 1.0) relative mRNA expression was significantly increased, in agreement with previous literature [33], suggesting that immune cells had infiltrated these tissues at day 3 in the MIA model (Figure 6B, E) However, no significant changes in the expression of either cell marker was found in the subchondral bone (Figure 6C, F).

**Figure 7:**
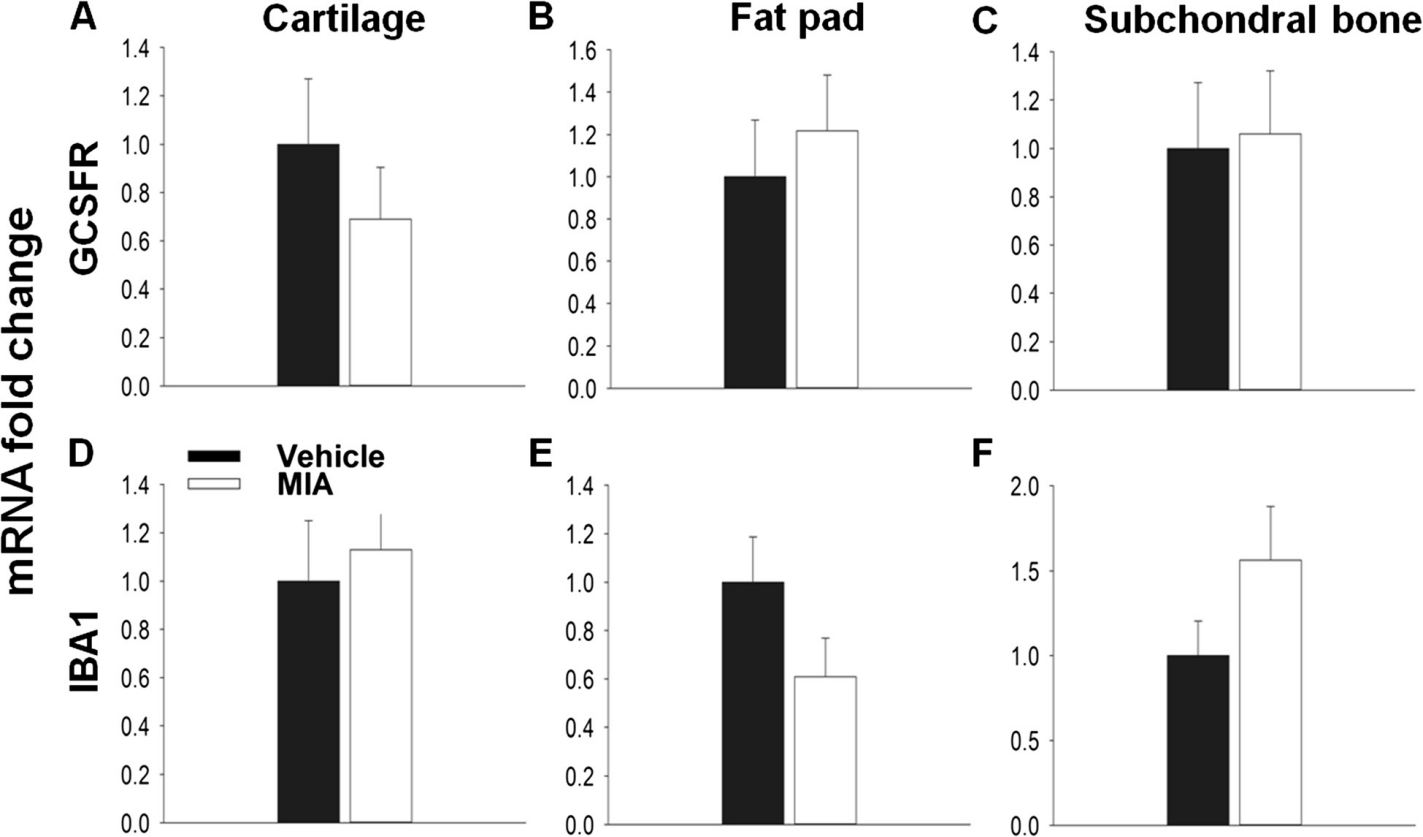
**Day 14: Expression of immune cell markers in the MIA model.** Relative changes in the transcript levels of GCSFR **(A-C)** and IBA **(D-F)** were measured in the MIA model compared to vehicle controls. No significant change was measured between MIA and controls in cell markers at both day 3 and 14 in all tissues **(A-F)**. T –Test **(B-F)**, Mann–Whitney Rank Sum Test **(A)**, *p < 0.05; n = 4. All data are expressed as mean ± SEM.

In the MIA model inflammation is lost by days 5 – 7 and by day 14 the model is thought to have entered a non-inflammatory state. In agreement with this, there is no difference in either GCSFR or IBA1 expression between vehicle and MIA treated animals at day 14 in any tissue, suggesting that the infiltration of these immune cells has subsided (Figure 7A-F).

## Discussion

The aim of this study was to measure the expression of inflammatory genes as possible mediators of chronic joint pain in the MIA model. Using the femorotibial joint tissues of cartilage, subchondral bone and fat pad, we found that many of these factors, in particular chemokines, were up-regulated at time points associated with pain-related behaviours. Although tissues were individually assessed, when data sets were evaluated it seemed that some factors, such as CCL2, 7 and 9 were consistently up-regulated, increasing the likelihood of them being pain mediators in OA.

Initially, pain-related behaviour was measured 3, 7 and 14 days post MIA injection. In agreement with previous reports, a significant ipsilateral weight bearing deficient was measured at each time point [22,33]. This effect peaked in the early inflammatory phase (day 3), consistent with other studies [27,33]. It is notable that both the intensity of pain-like behaviour and structural pathology are dependent on the dose of MIA [27,34]. One, two and three milligrams of MIA all produce robust pain-like behaviours, which are generally more profound as the dose is increased [19,20,34,35]. Activating transcription factor 3 (ATF3) expression in DRG neurons, a marker of peripheral nerve injury, is transiently expressed in the 1 mg MIA model [22]. However, the expression of ATF3 is more prominent and persistent following the injection of 2 mg of MIA and in agreement with nerve damage hallmarks there is a microgliosis in the corresponding area of the spinal cord, as well as a reduction in intra-epidermal nerve fibre density in the plantar hind paw skin [35,36]. Therefore the 1 mg MIA model, rather than being another model of nerve injury, likely induces persistent pain as a result of the degenerating joint. In addition, such as observed in OA patients [5], pain in this model can be attenuated by the intra-articular injection of lidocaine [34], suggesting that the abnormal sensory behaviour displayed by MIA animals after 1 mg of the chemical originates from joint tissues.

Following confirmation of pain-like behaviour, custom-made qPCR arrays were used to measure the expression of inflammatory mediators in the femorotibial joints at day 3 and 14. At day 3 the cartilage showed the largest transcriptional change compared to the fat pad and subchondral bone. OA pathology is thought to be initiated in the cartilage and in the MIA model the chondrocytes are initially targeted and undergo necrosis which is evident by day 1 [21]. Both cytokines and chemokines are involved in mediating this process [3,4]. For example, the chemokine CCL5, which was significantly up-regulated in the cartilage at day 3, has been shown to play a key role in the degradation of this tissue by activating chondrocytes to produce matrix metalloproteinases and other catabolic factors [37]. The macrophage marker IBA1 was also significantly increased. Therefore it is plausible to suggest that many inflammatory mediators are over-expressed in the cartilage at this stage either by atypical chondrocytes or by infiltrating immune cells. Importantly however, the cartilage is aneural and therefore pro-algesic mediators produced here would need to act on peri-articular tissues to cause pain. This inflammatory process is also prominent in the fat pad, which doubles in weight and is subjected to a significant monocyte and neutrophil infiltration [33], which was also measured here. In accordance a number of inflammatory mediators were up-regulated and included the chemokine CXCL5, which can recruit both neutrophils and macrophages *in vivo* and cause mechanical hypersensitivity when given to naïve rats [30]. Although not typically considered an important tissue in OA, the infrapatellar fat pad is highly innervated by nociceptive fibres [18] and is also a good source of cytokines and chemokines [38]. Interestingly, it has been suggested that the fat pad is one of the most pain-sensitive knee joint tissues in un-anaesthetised subjects [39] and it is of note that in a small cohort of OA sufferers 6 months after total joint replacement, more patients reported total pain relief if they also had the fat pad removed [40]. Therefore mediators produced here might be particularly important in causing OA pain. The degradation of the joint is also characterised by the remodelling of the underlying bone. Although histopathological changes in this tissue at day 3 have not previously been noted, we do find that on a transcriptional level, significant changes do occur.

At day 14, similar to day 3, the cartilage again displayed the greatest change in terms of mediator expression compared to the other tissues. In the fat pad at this stage no increase was measured for neutrophil or macrophage markers, in agreement with previous findings indicating that inflammation and immune cell infiltrate subside by day 14 [33]. Although inflammatory mediators were up-regulated, none of the changes were significant when compared to vehicle treated animals. At this point in the MIA model, the subchondral bone has begun to undergo significant remodelling with obvious bone resorption [19,21,22]. Therefore it is surprising that fewer mediators were dysregulated in this tissue at day 14. It is probable that a large transcriptional regulation is occurring in the subchondral bone, potentially comprising genes not measured by these qPCR array cards.

The aim of this study was to identify new putative pain mediators in chronic joint pain. By combining data sets the most prominently up-regulated genes were the chemokines CCL2, 7 and 9. In this study we have chosen to concentrate on mRNA expression levels as a proxy to estimate changes in protein levels, since this allows for the accurate measurement of numerous factors from small tissue samples. However an increase in mRNA does not always directly correlate with an exact increase in protein levels [31]. Here we measured a large and statistically significant increase in CCL2 mRNA in the cartilage 14 days after MIA injection. When looking at protein levels, again a large increase was measured. However due to high variability in the MIA group this was not significant. CCL2 is a secreted protein and as well as acting in the cartilage is it likely released into the joint capsule. The rate of this diffusion might be one reason for the increased variability seen. CCL2 protein was also measured in the subchondral bone and fat pad and found to be significantly increased compared to controls. This is in contrast to mRNA where no significant increases were found. One worry with measuring mRNA, particularly in this study, is that the large increases found in mRNA expression would not be mirrored on the protein level. Here we find the opposite. For the subchondral bone a strong trend for increased mRNA was found for CCL2 at day 14 which narrowly failed to reach statistical significance. However for the fat pad there was no increase in CCL2 mRNA. The increase in CCL2 protein is most likely the result of CCL2 protein being secreted into the fat pad from another tissue source. Although these data do not directly correlate, we find that on both an mRNA and protein level CCL2 is significantly up-regulated in the MIA treated joint at day 14.

As mentioned, a number of chemokines have been implicated in modulating pain processing and one possible mechanism is via the recruitment of immune cells. Previous work has shown that blocking the effects of chemokines in inflammatory pain models can reduce both neutrophil and macrophage infiltration and reduce pain-related hypersensitivity [30,41]. CCL2, which has been shown to have pro-nociceptive properties [14,42,43] can recruit immune cells [44]. It seems that CCL2 is responsible for macrophage recruitment into the injured nerve [45] and neuropathic pain can be attenuated by reducing their infiltration [46]. Therefore immune cells might contribute to OA pain particularly in the early inflammatory phase. Chemokines can also regulate the function of immune-related cells. For example CCL9 and its receptor CCR1 are the major chemokine ligand and receptor pair expressed by osteoclasts [47]. Osteoclast numbers increase in the later stages of the MIA model and their activity is responsible for bone resorption [21,34]. It is therefore intriguing that osteoclast activity has been implicated in pain-like behaviour in the MIA model [34] as well as in other pain models with bone pathology [48].

The contribution of immune cells to pain-related behaviours in the later stages of the MIA is unclear. At day 14 joint swelling has diminished, NSAIDs do not seem to affect pain behaviours [22] and there is no infiltration of immune cells. On the contrary, the increase in chemokine expression is still present and particularly in the case of the cartilage, even greater. The resolution of inflammation is regulated by many factors and these might act to prevent the action of chemokines on certain inflammatory cells. For example, one group of pro-resolving factors known as resolvins can act to prevent the expression of certain chemokine receptors by immune cells [49], thus preventing the ability of chemokines to recruit such cells. Instead chemokines might now act via a direct mechanism to cause pain since a number of members have been shown to induce calcium transients in cultured DRG neurons [50]. Recently, in a mouse model of surgically-induced OA, the increased expression of CCR2, the receptor for CCL2, was found in DRG neurons and these cells increased their responsiveness to ligand application *in vitro*[51]. Similar findings have been seen in nerve-injury models [52-55] and ATF3 expression is seen transiently in the DRG after 1 mg of MIA [22]. More in depth *in vitro* analysis has found that CCL2 can increase the activity of the sodium channel subunit Nav1.8 in DRG neurons [56] and Nav1.8 antagonists can reduce the firing rate of joint afferents and pain-related behaviours in the MIA model [57]. Therefore it seems possible that CCL2 produced in the joint could induce pain-related hypersensitivity by the direct sensitisation of sensory fibres. In agreement with both indirect and direct actions, CCR2 null mice do not develop movement induced pain following surgical induction of OA [51].

As mentioned CCL9, the most up-regulated factor in the highly innervated bone, acts through the CCR1 receptor [58]. The expression of this receptor has been found on DRG neurons [59]. Ligands acting on this receptor can either sensitise TRPV1 [59] or desensitise opioid receptors [60], in this way helping to induce or maintain a state of pain-related hypersensitivity. CCL7 was the top combined ranked chemokine in terms of up-regulation in the selected joint tissues of the MIA model. It is both similar in structure and function to CCL2 [61,62] and can also act through CCR1. Therefore it would be particularly interesting to see whether either CCL7 or CCL9 could act directly by looking at acute calcium responses in cultured DRG neurons from both naïve and MIA animals following their application.

The greatest up-regulation in chemokines was found in the cartilage at day 14 suggesting that a direct action would be unlikely since the cartilage is devoid of sensory nerve fibres. However, in OA patients progressive changes within the joint allow sensory nerve fibres to innervate the cartilage [15] and therefore chemokines produced by this tissue could now act directly on these fibres to cause pain. This innervation initially requires the vascularisation of the cartilage and chemokines have a well-defined role in angiogenesis [63] where blood vessels follow chemotactic gradients to vascularise tissue. Therefore the angiogenic properties of chemokines might represent an additional mechanism by which pain is facilitated in OA, particularly since neurovascularisation of cartilage as well as other articular tissues has been implicated in causing OA pain [64].

Evidence from clinical studies suggests that the persistent pain associated with OA has a strong peripheral component, most likely as a result of mediators acting within the affected joint. Here we show that many inflammatory factors are up-regulated in the MIA model, identifying them as putative pain mediators of chronic joint pain. In particular, a group of chemokines were consistently up-regulated and represent good targets for future studies in the development of treatments for OA pain.

## Methods

### Animals

Experiments were performed using male Wistar rats (~250 g, Harlan) in accordance with the United Kingdom Home Office Animals (Scientific Prodcedures) Act 1986. Food and water was available *ad libitum* and animals were housed under standard conditions with a 12 hour light/dark cycle.

### MIA model induction

Animals were anaesthetised with 3.5% isoflurane and subjected to a single intra-articular injection of 1 mg MIA (Sigma) based on previous literature [22]. Prior to injection, hair was removed from around the knee joint and cleaned with the use of an alcohol wipe. Doses of MIA were made up fresh in 20 μl of sterile physiological saline solution and administered with the use of a Hamilton syringe (Hamilton) through the infra-patellar ligament into the left knee joint capsule. Control animals received an intra-articular injection of physiological sterile saline alone.

### Behavioural testing

Weight bearing asymmetry was used as a measure of primary pain-related hypersensitivity. The weight borne on each hind limb was recorded with the use of an incapacitance meter (Linton Instruments). Rats were placed in a Perspex box and positioned so that their both hind paws were placed on force transducer pads. Once animals were settled and in the correct position, a reading of their weight distribution was taken. This reading was averaged over a 3 second period and the output produced an individual measurement of how much weight was borne on the ipsilateral and contralateral hind limbs. This method was repeated three times and the results averaged for each time point. Results were calculated as the percentage difference in weight distribution (Percentage difference = (ipsilateral/contralateral) x 100). Animals were trained for 1–2 weeks prior to MIA injections and baseline readings were obtained. All behavioural testing was performed blind.

### Tissue dissection and RNA extraction

Fat pad, cartilage and subchondral bone (containing underlying bone marrow) was removed from the femorotibial joint of both MIA and vehicle treated animals at 3 and 14 days post intra-articular injection. Rats were terminally anaesthetised and then transcardially perfused with cold physiological saline (0.9% Sodium chloride in dH_2_O). Following hair removal, the skin was cut to expose the femorotibial joint. The infrapatellar ligament, to which the fat pad is attached, was cut at the femoral head and dissected away from the joint capsule. Subsequently the fat pad was separated from the ligament. The knee joint capsule was opened by cutting the remaining cruciate and collateral ligaments. Cartilage was carefully removed from the tibial plateau and femoral condyles. The subchondral bone was taken from the tibia. Once removed each tissue was washed in sterile saline and snap frozen in liquid nitrogen and stored at -80°C. Tissue samples were then homogenised and total RNA obtained using a ‘hybrid’ method of phenol extraction (Trizol, Invitrogen) and column purification (RNeasy, Qiagen). This helped to achieve the extraction of high-quality RNA without a significant drop in yield (all 260:280 ratios were in the range of 1.94-2.12). All samples were DNase (Qiagen) treated to prevent genomic contamination and an RNA 6000 Nano Chip (Agilent) was used to ensure sufficient RNA integrity (RINs 7–10) and concentration was determined using a spectrophotometer (NanoDrop 1000). RNA was subsequently synthesised into cDNA using the Superscript II reverse transcriptase kit (Invitrogen) by following the manufacturer’s protocol.

### qPCR array cards and conventional qPCR

Taqman qPCR array cards were custom-made and designed using the Applied Biosystem website (http://www.appliedbiosystems.com). Each 384 well card contained 4 sets of 96 different primer pairs which included 4 reference genes (glyceraldehyde 3-phosphate dehydrogenase (GAPDH), 18 s, beta-actin and hypoxanthine phosphoribosyltransferase 1 (HPRT1)). For each tissue type, samples contained cDNA from individual animals. Each cDNA samples was diluted with polymerase chain reaction (PCR) grade water and added in a 1:1 ratio to Taqman Universal master mix producing a final concentration of 1 ng/μl. Samples were fed into the appropriate loading ports (1 μl for each well) and prepared according to the manufacturer’s guidelines. Cards were placed into a 7900HT Fast Real-Time PCR system (Applied Biosystems). Expression of each transcript was measured using the delta delta quantification cycle (2-^ΔΔCq^) method and analysis was carried out using the ReadqPCR and NormqPCR R packages [65]. Relative expression changes in transcript levels are presented as a fold change (FC = MIA/Control). Undetermined values were given a Cq (quantification cycle) value of 38. However, for a given detector, if more than 50% of samples were undetermined in both groups no FC was calculated and the transcript was described as undetected.

To measure the relative expression changes in immune cell markers, individual reverse transcriptase quantitative PCR was performed using the Corbett Rotor-Gene 6000. Samples were processed in duplicate and amplified using the Roche Lightcycler mastermix containing SYBR green for the detection of real-time changes. Primers were designed using Primer blast (Table 4) and the efficiency of all primers was in the range of 0.8-1.2. Transcript levels were again measured using the 2^-ΔΔCq^ method normalised against 18 s. The relative mRNA expression is shown as the amount of transcript in the treated samples versus control. Primer sequences for both G-CSFR and IBA1 were previously published [30].

**Table 4:** Primer sequences

**Gene**	**Forward**	**Reverse**
**IBA1**	TCCCCACCTAAGGCCACCAGC	CGTCTCCTCGGAGCCACTGGA
**GCSFR**	GGAGGGCTGCGGGCAAATCA	GGGACCCGTCAGGCAGGTGA
**CCL2**	TGCTGTCTCAGCCAGATGCAGTTA	TACAGCTTCTTTGGGACACCTGCT
**CCL9**	TGGGCCCACCAGGAGGATGAA	TCTGTCGCATGTACGATCTGGGC
**18 s**	CTTAGAGGGACAAGTGGCG	GGACATCTAAGGGCATCACA

### Combined-ranking

Within each data set genes were ranked by FC, from the most up-regulated to the most down-regulated when compared to expression in control samples. The top up-regulated genes was given a rank value of 92 (the number of all target genes), then next gene a value of 91 etc. The most down-regulated was given a value of 1 and those undetected genes were given a rank value of 0. To get an idea of gene regulation across different data sets an average of rank values was calculated to give a combined-rank value (CRV) for each gene. Genes were then ordered by CRV to show which genes were consistently up-regulated across tissues and time points in the MIA model.

### Protein extraction and ELISA

Tissue samples (cartilage, subchondral bone and fat pad) were dissected as described above. Since the amount of cartilage obtained at day 14 in the MIA is significantly reduced, samples were pooled in an effort to increase total protein concentrations. Following storage at -80°C, each tissue was washed and then homogenised in lysis buffer (phosphate buffered saline (PBS), 12.5 mM ethlenediaminetetraacetic (EDTA), 1:100 protease inhibitors (Sigma)). EMPIGEN (Sigma) was added to the homogenate (0.2%). Samples were then vortexed for 1 hour, centrifuged and supernatants collected. Total protein concentration was determined using a spectrophotometer (NanoDrop 1000). The amount of CCL2 protein was quantified using the Quantikine ELISA kit (R & D Systems) and by following the manufacturer’s instructions. For each sample of each tissue type 50 ul of neat lysate was used in duplicate and CCL2 protein concentration was determined against a standard curve. ELISA data is shown normalised to 1 mg/ml of the total protein loaded.

### Statistical analysis

For Taqman array cards, statistical significance was calculated by running t-tests in R (two-sided, Welch’s *t*-test) on the ΔCq values (ΔCq = gene of interest transcript Cq – reference gene Cq). To control for multiple hypothesis testing, the p values were adjusted using the FDR correction as proposed by Benjamini and Hochberg [66]. All other statistical analysis was carried out using the SigmaStat software. Where the data was not normally distributed and had unequal variance, the Mann–Whitney rank sum test was used to compare groups.

## Competing interests

The authors declare that they have no competing interests.

## Authors’ contributions

SBM and DLHB conceived of and coordinated the study. JMD carried out the animal surgery, processed tissue and performed conventional and array RT-qPCR experiments. JMD and HK carried out behavioural experiments and dissected tissue. JMD and JRP analysed RT-qPCR array data. SBM, DLHB, and JMD designed experiments. SBM and JMD wrote the manuscript. All authors read and approved the final manuscript.

## Supplementary Material

Additional file 1: Table S1 FC values for all inflammatory mediators measured in Cartilage, Subchondral bone and fat pad at both day 3 and 14. Click here for file

Additional file 2: Figure S1 Validation of CCL2 and CCL9 mRNA expression up-regulation in the MIA model. Relative changes in the transcript levels of CCL2 (A-C) and CCL9 (D-F) were measured in cartilage at day 3 and 14 (A&D and B&E, respectively) and subchondral bone at day 14 (C&F) in the MIA model and compared to vehicle controls using conventional QPCR. No significant increase was measured for CCL2 in the cartilage at day 3 (A) and subchondral bone at day 14 (C). A significant increase was found in CCL2 expression in the cartilage of MIA treated animals at day 14 compared to controls (B). A significant increase in CCL9 expression was measured for cartilage at day 3 and 14 and the subchondral bone at day 14 (D, E F). T –Test (A, C, D, F), Mann–Whitney Rank Sum Test (B, E), *p < 0.05; n = 4. All data are expressed as mean ± SEM. Click here for file
