# Supplementary figures and images for: Chemokine expression in peripheral tissues from the Monosodium Iodoacetate model of chronic joint pain
Source: Mol Pain. 2013 Nov 8;9:57. doi: 10.1186/1744-8069-9-57 (PMC3835139; doi:10.1186/1744-8069-9-57)

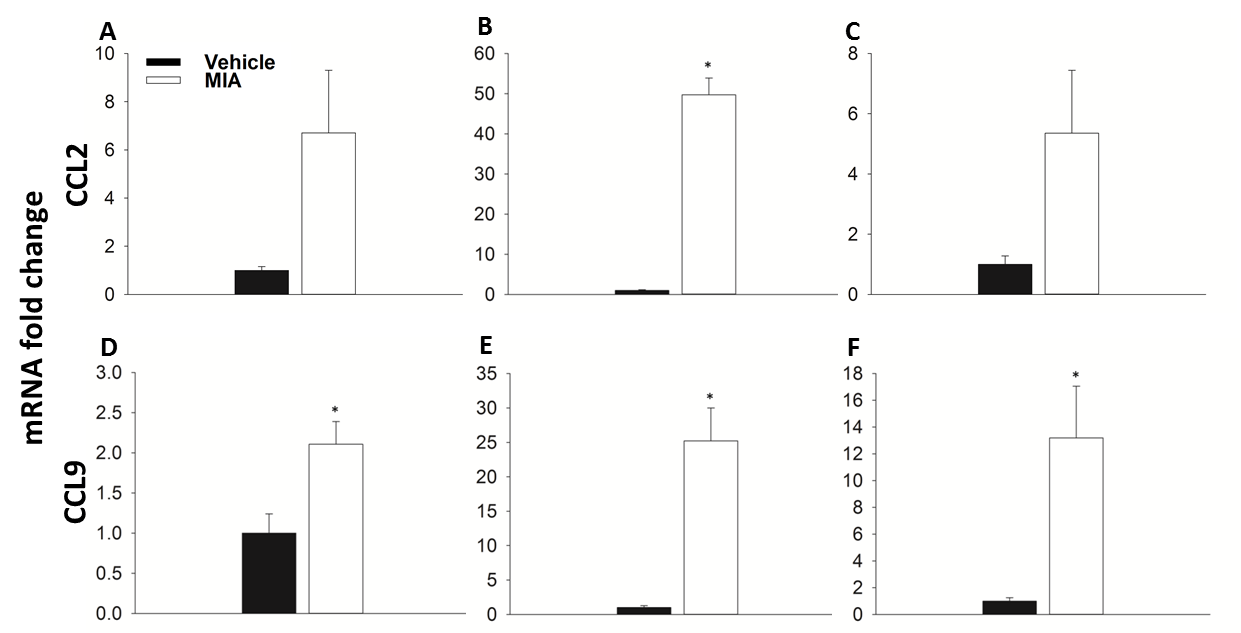

Supplement: Additional file 2: Figure S1 — Validation of CCL2 and CCL9 mRNA expression up-regulation in the MIA model. Relative changes in the transcript levels of CCL2 (A-C) and CCL9 (D-F) were measured in cartilage at day 3 and 14 (A&D and B&E, respectively) and subchondral bone at day 14 (C&F) in the MIA model and compared to vehicle controls using conventional QPCR. No significant increase was measured for CCL2 in the cartilage at day 3 (A) and subchondral bone at day 14 (C). A significant increase was found in CCL2 expression in the cartilage of MIA treated animals at day 14 compared to controls (B). A significant increase in CCL9 expression was measured for cartilage at day 3 and 14 and the subchondral bone at day 14 (D, E F). T –Test (A, C, D, F), Mann–Whitney Rank Sum Test (B, E), *p < 0.05; n = 4. All data are expressed as mean ± SEM. [file 1744-8069-9-57-S2.tiff]
